# Lucky Guy Knife Assault

**Published:** 2013-09-01

**Authors:** Farideh Roshanali, Mohammad Hossein Mandegar, Bahieh Moradi

**Affiliations:** 1 Department of Echocardiography, Dey General Hospital, Tehran, IR Iran; 2 Department of Cardiothoracic Surgery, Shariati General Hospital, Tehran University of Medical Science, Tehran, IR Iran

**Keywords:** Chest, Trauma

## Abstract

We reported a young male with knife assault to his chest. He was transferred to the hospital without manipulating the knife. He was completely conscious and had sinus tachycardia and regular breathing. Emergency thoracotomy was performed and the knife was removed. No organ was damaged. This case presentation showed that in this kind of trauma, it is mandatory not to manipulate the penetrating foreign body during the transfer to the hospital.

## 1. Introduction

Approximately 13 people a day are currently admitted to hospitals for treatment after being stabbed. Among these individuals, 42% have head, neck, or thorax injuries (1). The assault using a knife is a common problem in many countries (2). Therefore, finding ways to increase the survival rate in these conditions seems to be of great importance.

## 2. Case Report

**Figure 1.**
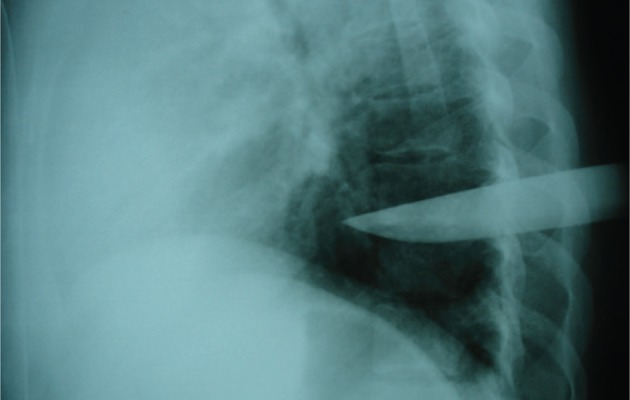
Chest Radiography in Lateral Position; the Tip of Deep Penetrated Knife Is Closed to Descending Aorta

**Figure 2.**
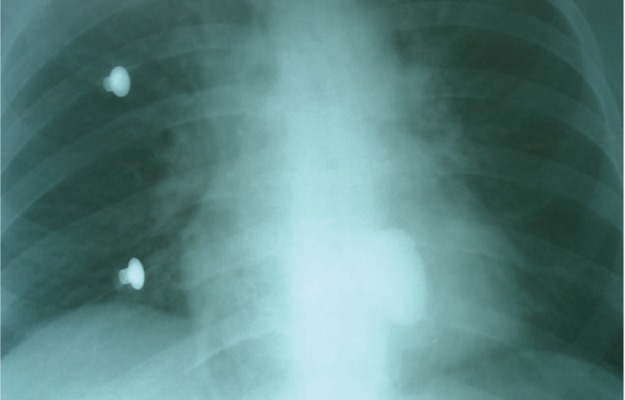
Chest Radiography in Supine Position (AP); the Assaulted Knife in the Chest

A 24-year-old man was brought to our emergency department with a knife in his chest (Figure 1, 2). He was completely oriented with mild bleeding in the trauma site. Physical examination revealed HR=130 beats per minute is correct BP=90/60 mmHg, regular breathing, and cold sweating. The patient was urgently rushed to the operating room where thoracotomy was performed and the knife was removed. No organ, not even the lung tissue, was damaged by the knife (tip of the knife touched the descending aorta without entering it and there was scratch on the adventitia of the aorta). He was discharged from the hospital alive after two weeks.

## 3. Discussion

The number of recorded hospital admissions from stabbing assaults has increased in the recent years (1). The risk of death appears to depend mostly upon the injuries sustained and, to a lesser extent, upon other factors, such as alcohol consumption and the presence of a bystander capable and willing to request emergency medical assistance (2). For better evaluation and management of stabbing trauma, many scoring systems, such as ISS and ICD-9(3, 4), have evolved; however, the care provided to the trauma patients needs to be improved. Nevertheless, improving the hospital treatment for those assaulted with a knife does not appear to be much potent to save lives. Instead, greater focus needs to be placed upon the rapid transfer to hospitals and restricting the possession and use of knives. Overall, the present report showed that it is vitally important to know that the key maneuver in this kind of trauma is not to manipulate the penetrating foreign body during the transfer to the hospital.
